# Comparison of Testosterone Levels in Patients With and Without Type 2 Diabetes

**DOI:** 10.7759/cureus.16288

**Published:** 2021-07-09

**Authors:** Naina Kumari, Anoosha Khan, Usman Shaikh, Kimberly Lobes, Deepak Kumar, FNU Suman, Naila S Bhutto, Faryal Anees, Simra Shahid, Amber Rizwan

**Affiliations:** 1 Internal Medicine, Dow University of Health Sciences, Karachi, PAK; 2 Internal Medicine, Liaquat National Hospital and Medical College, Karachi, PAK; 3 Internal Medicine, Jinnah Sindh Medical University, Karachi, PAK; 4 Internal Medicine, Peoples University of Medical Health Sciences for Women, Nawabshah, PAK; 5 Internal Medicine, Chandka Medical College, Chandka, PAK; 6 Family Medicine, Jinnah Post Graduate Medical Center, Karachi, PAK

**Keywords:** testosterone, type 2 diabetes, diabetes mellitus, testosterone replacement therapy, hypogonadism

## Abstract

Introduction

Hypogonadotropic hypogonadism is a common disorder associated with type 2 diabetes. Hypogonadotropic hypogonadism in type 2 diabetic patients requires further assessment to understand the etiology, and the possible consequences, complications, and treatment This study aims to highlight the testosterone level in type 2 diabetes mellitus (DM). Moreover, it further emphasizes the association of testosterone with the duration of DM.

Materials and method

This case-control survey was conducted from September 2020 to March 2021 in the outpatient department of internal medicine in a tertiary care hospital in Pakistan. The experiment group included 200 diabetic male participants aged between 30 and 69 years. In the control group, 200 participants without DM were enrolled in the study. The venous blood sample was collected via phlebotomy and sent to the laboratory to test for total testosterone level.

Results

The mean total testosterone level was significantly lower in diabetic patients compared to the non-diabetic patients (8.9 ± 5.1 mmol/L vs. 14.1 ± 7.2 mmol/L; p-value: <0.0001) and the prevalence of androgen deficiency was significantly higher in diabetic patients compared to non-diabetic patients (45.5% vs. 20.5%; p-value: <0.00001). For each age group, the mean total testosterone level was significantly higher in the diabetic group compared to the non-diabetic group. There was a significant decline in mean total testosterone level as the duration of diabetes increased (p-value: 0.01).

Conclusion

Strong interlink between type 2 DM and low testosterone level has once again highlighted the importance of a broader approach toward men presenting in the diabetic clinic and provided a huge ground for prescribing testosterone replacement therapy in hypogonadal men with DM.

## Introduction

Hypogonadotropic hypogonadism is a common disorder associated with type 2 diabetes. Hypogonadotropic hypogonadism in type 2 diabetic patients requires further assessment to understand the etiology, and the possible consequences, complications, and treatment [1]. Lower testosterone levels were observed in 25%-50% of men with type 2 diabetes mellitus (DM) [1].

An Australian study including 580 elderly, obese men with type 2 diabetes was conducted, proving that 43% had low total testosterone levels and 53% had low calculated free testosterone levels [2]. Serum testosterone showed an inverse association with age and BMI in these men; however, low testosterone levels were not restricted to these factors along with low circulating testosterone in 20% young and 40% lean men [3]. A meta-analysis of 20 cross-sectional studies, including 850 type 2 diabetic men, proved that total testosterone levels were adequately reduced in diabetics [4]. Subnormal testosterone levels are comparatively uncommon in men with type 1 DM. In another cross-sectional study of 350 Finnish men, sex hormone-binding globulin (SHBG) was proved to have an association with insulin resistance independent of testosterone, which nullified the association of total testosterone with insulin resistance [5]. A Mendelian randomization study proved that SHBG-germline variants were predicted to be a risk factor of type 2 DM [6]. However, men with low testosterone levels have a twofold risk of development of metabolic or type 2 DM [7].

This topic of testosterone and DM is understudied in developing countries. This study aims to highlight the testosterone level in type 2 diabetics. Moreover, it further emphasizes the association of testosterone with the duration of DM.

## Materials and methods

This case-control survey was conducted from September 2020 to March 2021 in the outpatient department of internal medicine in a tertiary care hospital in Pakistan. The experiment group included 200 diabetic male participants aged between 30 and 69 years. In the control group, 200 participants without DM were enrolled in the study. Participants were enrolled via consecutive convenient non-probability sampling. Ethical review board approval was taken before enrolling participants in the study. Exclusion criteria included renal disease, chronic liver disease, malignancies, and androgen therapy, as they may negatively impact testosterone levels.

After informed consent, the participant’s age, BMI, and duration of DM was noted in a self-structured questionnaire. The venous blood sample was collected via phlebotomy and sent to the laboratory to test for total testosterone level. The sample was collected in the morning to avoid variation in testosterone levels. Participants with total testosterone less than 8 mmol/L were labeled as androgen deficient [8].

Statistical Package for Social Sciences® software, version 22.0 (SPSS; IBM Corp., Armonk, NY, USA) was used for data analysis. For numerical variables, data were expressed as mean ± standard deviations. Frequencies and percentages were used for categorical variables. Mean testosterone level was compared using an independent t-test. Chi-square was used to compare the prevalence of androgen deficiency between the two groups. ANOVA test was used to compare mean testosterone levels for different groups stratified based on the duration of DM. Data were stratified based on age group and duration of DM. A p-value of less than 0.05 indicated that there is a difference between both groups and the null hypothesis is not valid.

## Results

Two groups were comparable in terms of their age distribution, smoking history, and BMI (Table 1).

**Table 1:** Comparison of characteristics of participants BMI: body mass index, kg/m^2^: kilogram per square meter

Characteristics	Diabetic (n=200)	Non-diabetic (n=200)	P-value
Age group in years			
30-39	17 (8.5%)	15 (7.5%)	0.9
40-49	35 (17.5%)	40 (20.0%)	
50-59	101 (50.5%)	97 (48.5%)	
60-69	47 (23.4%)	48 (24.0%)	
Smoking			
Yes	92 (46.0%)	99 (49.5%)	0.48
No	108 (54.0%)	101 (50.5%)	
BMI more than 25 kg/m^2^			
Yes	57 (28.5%)	54 (27.0%)	0.11
No	143 (71.5%)	146 (73.0%)	

The mean total testosterone level was significantly lower in diabetic patients compared to the non-diabetic patients (8.9 ± 5.1 mmol/L vs. 14.1 ± 7.2 mmol/L; p-value: <0.0001). The prevalence of androgen deficiency was significantly higher in diabetic patients compared to non-diabetic patients (45.5% vs. 20.5%; p-value: <0.00001) (Table 2).

**Table 2:** Comparison of mean total testosterone level and androgen deficiency in both groups mmol/L: millimoles per liter

Values	Diabetic (n=200)	Non-diabetic (n=200)	P-value
Mean total testosterone level (mmol/L)	8.9 ± 5.1	14.1 ± 7.2	<0.0001
Androgen deficiency	91 (45.5%)	41 (20.5%)	<0.00001

For each age, group, the mean total testosterone level was significantly higher in the diabetic group compared to the non-diabetic group (Table 3).

**Table 3:** Age-wise comparison of mean serum testosterone levels in both groups mmol/L: millimoles per liter

Age group (in years)	Mean total testosterone level (mmol/L)		P-value
	Diabetic (n=200)	Non-diabetic (n=200)	
30-39	14.2 ± 6.1	20.1 ± 7.5	0.0018
40-49	11.6 ± 5.7	18.2 ± 7.1	<0.0001
50-59	8.8 ± 5.1	14.5 ± 6.2	<0.0001
60-69	6.8 ± 4.1	9.9 ± 5.9	0.0038

There was a significant decline in mean total testosterone level as the duration of diabetes increased (p-value: 0.01). The mean total testosterone level of participants with a duration of diabetes less than five years was 9.7 ± 5.8 mmol/L. The mean total testosterone level of participants with a duration of diabetes for more than 10 years was 6.8 ± 4.6 mmol/L (Table 4).

**Table 4:** Correlation of duration of diabetes and mean serum testosterone levels mmol/L: millimoles per liter

Duration of diabetes	Mean total testosterone level (mmol/L)	P-value
Less than 5 years (n=61)	9.7 ± 5.8	0.01
Between 5 and 10 years (n=81)	8.7 ± 5.4	
More than 10 years (n=58)	6.8 ± 4.6	

## Discussion

Type 2 DM is an element of danger for impairing sex steroid status and is a risk factor for developing testosterone deficiency. Marked decline in the serum testosterone has been reported in a significant number of men with type 2 DM [9], favoring the results of our study that there is a remarkable decline in the total serum testosterone level in patients with diabetes than in patients without diabetes i.e. (8.9 ± 5.1 mmol/L vs. 14.1 ± 7.2 mmol/L; p-value: <0.0001). The mean total testosterone was found to be significantly reduced for each age group in diabetics compared to the non-diabetics. Likewise, androgen deficiency was higher in diabetics than non-diabetics. Inverse relation of total serum testosterone level and duration of diabetes have also been noticed. The mean total testosterone level of participants with the duration of diabetes less than five years was 9.7 ± 5.8 mmol/L. The mean total testosterone level of participants with a duration of diabetes for more than 10 years was 6.8 ± 4.6 mmol/L. In contrast to our study, in the Massachusetts Male Aging Study (MMAS) cohort, a 0.4% reduction of total serum testosterone level per year was found in both healthy population and men with chronic diseases, including type 2 DM, but the decline in diabetic subjects was 10%-15% lower compared to age-matched healthy subjects [10]. Reports have established that at least 25% of diabetic patients are testosterone deficient with abnormal luteinizing hormone (LH) and follicle-stimulating hormone (FSH) concentrations [11]. A study from Jordan enrolled 1,089 type 2 diabetic men between age 30 and 70 years showed the 36.5% prevalence of low serum testosterone level, i.e. <3 ng/mL, with 29% of patients exhibiting symptoms of androgen deficiency. It has also been concluded that mean gonadal hormone level decreases with increasing age and duration of diabetes [12]. One-third of US men aged 65 and older have DM with almost the same percentage of serum testosterone deficiency [13]. A meta-analysis by Ding et al. showed a consistent decline in total serum testosterone in the diabetic population compared to the men without diabetes, with a mean pooled difference of −2.66 nmol/L [95% confidence interval, −3.45 to −1.86] [4].

This coexistence of DM and testosterone deficiency in men arise the vigorous debate whether it is the low level of testosterone leading to metabolic dysfunction or it is just the biomarker coexisting with diabetes, or low total serum testosterone is a consequence of prolonged diabetes [14]. According to recent studies, both Asian and Caucasian men with low serum testosterone levels are more vulnerable to developing metabolic syndrome, including type 2 DM with adverse cardiovascular clinical outcomes [15].

There is consistent evidence that low serum testosterone is associated with future diabetes or predicts diabetes, but the association is moderate with a 2-3 nmol/L decline in total testosterone. It has also been found that association is stronger for total than free serum testosterone, showing a strong role of SHBG. This association is weakened by adjusting waist circumference showing a strong impact of abdominal fat on total serum testosterone [16]. The bulk of evidence has suggested that very low testosterone increases insulin resistance [17]. Testosterone is essential for regulating insulin by regulating adipocytes and myocytes and by enhancing catecholamine-induced lipolysis; hence, reduced fat mass and increased muscle mass are the changes required for decreasing insulin resistance [18].

In contrast, other studies concluded a strong correlation of insulin-dependent diabetes and its effect on the function of Leydig cells and testosterone production because of the absence of stimulatory effect of insulin on these cells and an insulin-dependent decrease in FSH [19].

Hence, the strong association between the two provides a massive ground for trying testosterone replacement therapy in hypogonadal men with type 2 DM and men with symptoms of androgen deficiency and whether it impacts insulin resistance in any way. A study shows patients who received daily treatment with 120 mg oral testosterone undecanoate for three months showed improved glucose homeostasis and body composition, i.e. decreased visceral obesity, and improved symptoms of androgen deficiency, including erectile dysfunction. In these men, the benefit of testosterone supplementation therapy exceeds the correction of symptoms of androgen deficiency and also includes glucose homeostasis and metabolic control [20].

To the best of our knowledge, this is the first study in a local setting that studied the association between testosterone and diabetes. However, there are few limitations as well. First, the study was conducted in a single center, so the sample size was less diverse. The second limitation was because of the unavailability of a kit to test free testosterone, total serum testosterone was used.

## Conclusions

Our study indicates an association between type 2 diabetes and a low level of testosterone. Our study has once again highlighted the importance of a broader approach toward men presenting in the diabetic clinic and provided a huge ground for prescribing testosterone replacement therapy in hypogonadal men with DM. Further studies should be conducted to reproduce more data in support of evidence of improved glucose homeostasis and gonadal function in men with type 2 DM.
